# Annotating the human genome with Disease Ontology

**DOI:** 10.1186/1471-2164-10-S1-S6

**Published:** 2009-07-07

**Authors:** John D Osborne, Jared Flatow, Michelle Holko, Simon M Lin, Warren A Kibbe, Lihua (Julie) Zhu, Maria I Danila, Gang Feng, Rex L Chisholm

**Affiliations:** 1 Department of Microbiology, University of Alabama at Birmingham, Birmingham, AL 35294, USA; 2 The Biomedical Informatics Center, Robert H. Lurie Comprehensive Cancer Center, Northwestern University, Chicago, IL 60611, USA; 3 Department of Preventive Medicine, Robert H. Lurie Comprehensive Cancer Center, Northwestern University, Chicago, IL 60611, USA; 4 The Center for Genetic Medicine, Robert H. Lurie Comprehensive Cancer Center, Northwestern University, Chicago, IL 60611, USA; 5 Program in Gene Function and Expression, University of Massachusetts Medical School, Worcester, MA 01605, USA; 6 Division of Clinical Immunology and Rheumatology, University of Alabama at Birmingham, Birmingham, AL 35294, USA

## Abstract

**Background:**

The human genome has been extensively annotated with Gene Ontology for biological functions, but minimally computationally annotated for diseases.

**Results:**

We used the Unified Medical Language System (UMLS) MetaMap Transfer tool (MMTx) to discover gene-disease relationships from the GeneRIF database. We utilized a comprehensive subset of UMLS, which is disease-focused and structured as a directed acyclic graph (the Disease Ontology), to filter and interpret results from MMTx. The results were validated against the Homayouni gene collection using recall and precision measurements. We compared our results with the widely used Online Mendelian Inheritance in Man (OMIM) annotations.

**Conclusion:**

The validation data set suggests a 91% recall rate and 97% precision rate of disease annotation using GeneRIF, in contrast with a 22% recall and 98% precision using OMIM. Our thesaurus-based approach allows for comparisons to be made between disease containing databases and allows for increased accuracy in disease identification through synonym matching. The much higher recall rate of our approach demonstrates that annotating human genome with Disease Ontology and GeneRIF for diseases dramatically increases the coverage of the disease annotation of human genome.

## Background

High throughput genomics technologies generate a vast amount of data. Determining the biologically and clinically significant findings of an experiment can be a daunting task. Applying functional knowledge to genomic data is one method that has been used to reduce data complexity and establish biologically plausible arguments. These methods rely on *a priori *definition of gene sets, and the results necessarily depend on the strength of the annotations [1,2]. Genome-wide annotation of gene function has garnered much attention and the comprehensive Gene Ontology (GO) Consortium annotations are widely used [3]. Few tools based on ontology are available for annotating genome-wide data with disease associations. The lack of ontology based disease annotation prevents the application of disease knowledge to genomic data, therefore hindering the discovery of gene-disease associations from high throughput genomics technologies.

Online Mendelian Inheritance in Man (OMIM), curated by the NCBI and Johns Hopkins University, is arguably the most widely used disease gene annotation database. Although the curation process provides highly detailed annotation and minimizes errors, there is a noticeable delay in updating. Furthermore, the vocabulary of OMIM is predominately text based, far from comprehensive, and is difficult to use [4-6]. It is simply not possible to download a list of diseases from OMIM and users have resorted to mining the Clinical Synopsis free text section of OMIM for disease discovery [7]. It also focuses on genetic diseases with classic Mendelian inheritance, thus eliminating the wide range of diseases resulting from more complicated environmental and genetic interactions.

Another source of gene-disease mappings from linkage studies is the "Genetic Association Database" (GAD) [4] which aims to "collect, standardize and archive genetic association study data". The structure of the classification system GAD used to classify its diseases is not apparent. Diseases are classified in 10 broad classes including an "other" class (some remain unclassified), an unknown number of broad phenotype classes below those and a further number of narrow phenotype classes. This lack of apparent ontology makes it hard to determine the number and types of diseases GAD contains. For example, searching for "Crohn's disease" returns 25 results but searching for "regional enteritis" returns no results.

Researchers have also used abstracts and titles from MEDLINE as a data source for inferring gene-disease associations [8,9]. Although a current and rich source of information, the free text form of MEDLINE abstracts presents difficulties for determining the context of the association between gene and disease [10]. This is particularly true when genes are identified by semantically ambiguous gene symbols which may or may not apply to a disease recognized in free text. For instance CAT can refer to the catalase gene or a feline animal, depending on the context.

A GeneRIF (Gene Reference Into Function) is a brief (up to 255 character) annotation to a gene in the NCBI database and contains gene specific information including disease associations. These entries are modifiable by NCBI users willing to provide their email address. Such a Wiki-type of resource offers low mapping error of gene symbols and allows a rapid update by the research community [11]. Despite this utility, GeneRIF has been infrequently considered as a data source for text mining, evidenced by the fact that only six papers indexed in pubmed contain the term "GeneRIF". One of these describes a data mining tool called MILANO, which counts occurrences of each GeneRIF annotated gene with user-defined terms selected from Medical Subject Headings (MeSH) and includes some disease terms [12]. The authors found GeneRIF superior to Medline, PubMatrix, BEAR GeneInfo, and MicroGenie, for identifying p53 affected genes. A more recent approach using conditional random fields to map a test set of geneRIFs to MeSH terms further validates geneRIFs as a comprehensive data source [13] for the human genome annotation.

To provide a comprehensive disease to gene annotation, we used the Disease Ontology (DO) [14] to identify relevant diseases in GeneRIFs. The Disease Ontology is a manually inspected subset of Unified Medical Language System (UMLS) and includes concepts from outside the UMLS disease/disorder semantic network including various cancers, congenital abnormalities, deformities and mental disorders. While many researchers have mapped diseases to MeSH terms [8,15-17] or OMIM [5,7] the Disease Ontology is larger and should therefore provide greater disease coverage. The hierarchal structure also allows more general disease terms to be distinguished from subclasses, in order to account for "over-mapping" of disease terms to a textually larger database. We used a thesaurus based approach (MetaMap Transfer tool, MMTx) for analyzing GeneRIFs with demonstrated success in studying clinically relevant terms [18].

## Results

### Mapping genes to Disease Ontology

**Figure 1:**
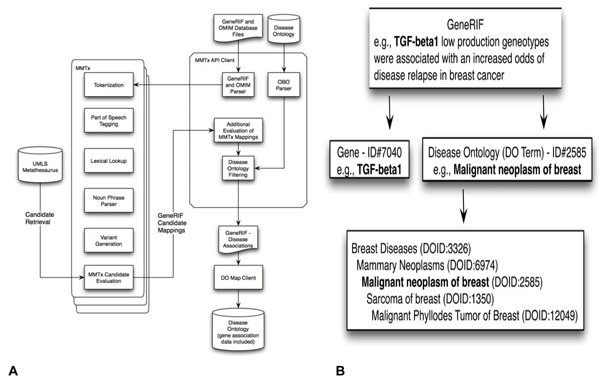
**Diagram of Disease Ontology annotation of the human genome**. A) MMTx was used to annotate GeneRIFs with the Disease Ontology (DO). B) An example GeneRIF suggests that Gene ID: 7040 is annotated with DOID:2585.

The process for mapping Disease Ontology (DO) terms to GeneRIFs is illustrated in Figure 1A. The Disease Ontology is a disease-focused comprehensive subset of Unified Medical Language System (UMLS) and outside terms structured as a directed acyclic graph, similar to the structure of the Gene Ontology (GO) from the GO Consortium. MetaMap Transfer tool (MMTx) was used to map the DO to GeneRIFs. These mappings are stored in the Open Biomedical Ontologies (OBO; ) format and can be manually edited using the open source graph editor DAGEdit . An example gene-disease association is shown in Figure 1B. The GeneRIF entry for TGFB1 links the gene to the DO term. The related Disease Ontology terms are also provided for the mapped DO term. In cases where multiple mappings of a gene are possible along a branch of the Disease Ontology tree, genes are mapped to the most specific disease term.

**Figure 2:**
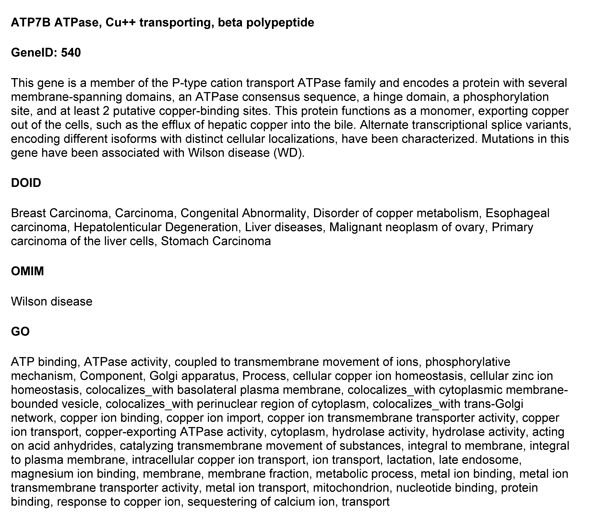
**Example Gene Annotation by DO, OMIM and GO**. **ATP7B ATPase, Cu++ transporting, beta polypeptide**. **GeneID: 540**. This gene is a member of the P-type cation transport ATPase family and encodes a protein with several membrane-spanning domains, an ATPase consensus sequence, a hinge domain, a phosphorylation site, and at least 2 putative copper-binding sites. This protein functions as a monomer, exporting copper out of the cells, such as the efflux of hepatic copper into the bile. Alternate transcriptional splice variants, encoding different isoforms with distinct cellular localizations, have been characterized. Mutations in this gene have been associated with Wilson disease (WD). **DOID**. Breast Carcinoma, Carcinoma, Congenital Abnormality, Disorder of copper metabolism, Esophageal carcinoma, Hepatolenticular Degeneration, Liver diseases, Malignant neoplasm of ovary, Primary carcinoma of the liver cells, Stomach Carcinoma. **OMIM**. Wilson disease. **GO**. ATP binding, ATPase activity, coupled to transmembrane movement of ions, phosphorylative mechanism, Component, Golgi apparatus, Process, cellular copper ion homeostasis, cellular zinc ion homeostasis, colocalizes_with basolateral plasma membrane, colocalizes_with cytoplasmic membrane-bounded vesicle, colocalizes_with perinuclear region of cytoplasm, colocalizes_with trans-Golgi network, copper ion binding, copper ion import, copper ion transmembrane transporter activity, copper ion transport, copper-exporting ATPase activity, cytoplasm, hydrolase activity, hydrolase activity, acting on acid anhydrides, catalyzing transmembrane movement of substances, integral to membrane, integral to plasma membrane, intracellular copper ion transport, ion transport, lactation, late endosome, magnesium ion binding, membrane, membrane fraction, metabolic process, metal ion binding, metal ion transmembrane transporter activity, metal ion transport, mitochondrion, nucleotide binding, protein binding, response to copper ion, sequestering of calcium ion, transport. An example gene annotation is provided for ATP7B. The gene description, DOID, OMIM, and GO annotation descriptions are provided.

Disease ontology annotations of a human gene describe unique roles for genes in the context of disease, and are complementary to gene ontology annotations. The gene-disease mapping for ATP7B is provided as an example in Figure 2. The gene description is provided, along with DO, OMIM, and GO annotations. This example demonstrates that GeneRIF results in more disease associations than OMIM for this gene. Also note that the DO annotation uses a formal vocabulary of "hepatolenticular degeneration" instead of "Wilson disease".

**Figure 3:**
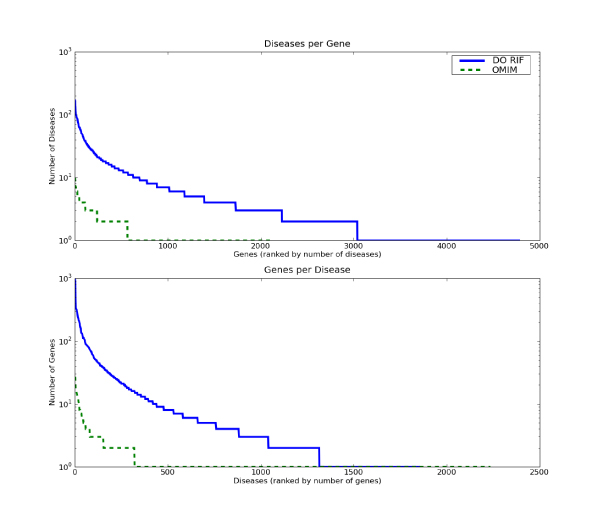
**Comparison of DO and OMIM Annotation**. A) The number of diseases per gene is plotted for the Disease Ontology (DO) analysis and OMIM. B) The number of genes per disease is plotted for the Disease Ontology (DO) analysis and OMIM.

To assess the gene-disease associations we identify, graphs illustrating the mapping of single genes to diseases as well as single diseases to genes are presented in Figure 3. Similar results from OMIM are reported on the graph as a point of reference. Plots of the number of diseases per gene (Figure 3a) and the number of genes per disease (Figure 3b) suggest the depth and coverage of DO annotation is higher than OMIM. In addition, both the number of diseases per gene and the number of genes per disease demonstrate scale-free properties, which have been observed in both biological networks and citation networks [19]. As such, there is no 'mode' or 'scale' as observed in a Gaussian distribution; the number of diseases per gene spreads from 195 to 1. The gene with the greatest number of disease associations is Interleukin 6 (IL6). Moreover, the result from the DO annotation suggests a 'rich get richer' phenomenon: the top 48 (1%) genes are implicated in 931 (50%) diseases.

Not surprisingly, several cancers including breast, prostate, liver, colon, and metastatic lesions have a larger number of genes associated with them (Table 1). Other non-cancer diseases implicated with many genes include other complex diseases including diabetes and rheumatoid arthritis. When we look at the genes associated with many diseases, the interleukins including IL1B, IL6, IL8, and IL10 are near the top of the list (Table 2). Biologically, this is likely due to the fact that inflammation is a common pathological consequence. In addition, matrix metalloproteinases MMP2 and MMP9, the cell growth and cell cycle regulators CDKN2A, BCL2, and EGFR, and Methylentetrahydrofolate reductase (MTHFR) genes are among the top genes associated with numerous disease conditions.

**Table 1:** First Ten Diseases ordered by the number of gene annotations

**DOID**	**Description**	**Number of Genes**
DOID:162	Cancer	943
DOID:462	Malignant Neoplasms	903
DOID:4241	Malignant neoplasm of breast	698
DOID:4766	Embryoma	620
DOID:10283	Malignant neoplasm of prostate	543
DOID:2619	Neoplasm Metastasis	386
DOID:9352	Diabetes Mellitus, Non-Insulin-Dependent	329
DOID:684	Primary carcinoma of the liver cells	326
DOID:7148	Rheumatoid Arthritis	320
DOID:1994	Carcinoma of the Large Intestine	313

**Table 2:** First Ten Genes ordered by the number of disease annotations

**Entrez ID**	**Gene Symbol**	**Gene Name**	**Number of Diseases**
3569	IL6	interleukin 6	168
4318	MMP9	matrix metallopeptidase 9	164
1956	EGFR	epidermal growth factor receptor	138
1029	CDKN2A	cyclin-dependent kinase inhibitor	138
4313	MMP2	matrix metallopeptidase 2	135
3586	IL10	interleukin 10	134
4524	MTHFR	5,10-methylenetetrahydrofolate reductase	123
3576	IL8	interleukin 8	121
596	BCL2	B-cell CLL/lymphoma 2	115
3553	IL1B	interleukin 1, beta	109

### Performance evaluation

For evaluation, we use two commonly used performance metrics in textual data retrieval, which are defined as follows:





From these formulas, we can see they are closely related to false positive and false negative rates that are used in other fields. A recall rate of 100% and a precision rate of 100% are of ideal situations. For disease annotation, we constructed a truth table of the Homayouni gene collection [20] manually using GeneRIF and OMIM text as a source.

For the Homayouni gene collection, there are 3879 GeneRIFs, with an average of 77.58 GeneRIFs per gene and a median of 22. On average our algorithm maps 18.8% of GeneRIFs to a disease, with recall and precision rates of 90.76% and 96.66% respectively (Table 3). Mapping the Homayouni gene collection to OMIM, however, results in a recall rate of 21.85% and precision of 98.46%. Since OMIM is a curated database, the slightly higher precision rate for OMIM compared to GeneRIF is not surprising. However, this small loss in precision by using OMIM is accompanied by a dramatically reduced recall rate.

**Table 3:** Estimation of recall and precision of disease annotation

	OMIM	GeneRIF
Recall	21.85	90.76
Precision	98.46	96.66

The Homayouni gene collection was used to estimation of recall and precision of gene mappings to the Disease Ontology.

### A network visualization based on the DO Annotation of the human genome

**Figure 4:**
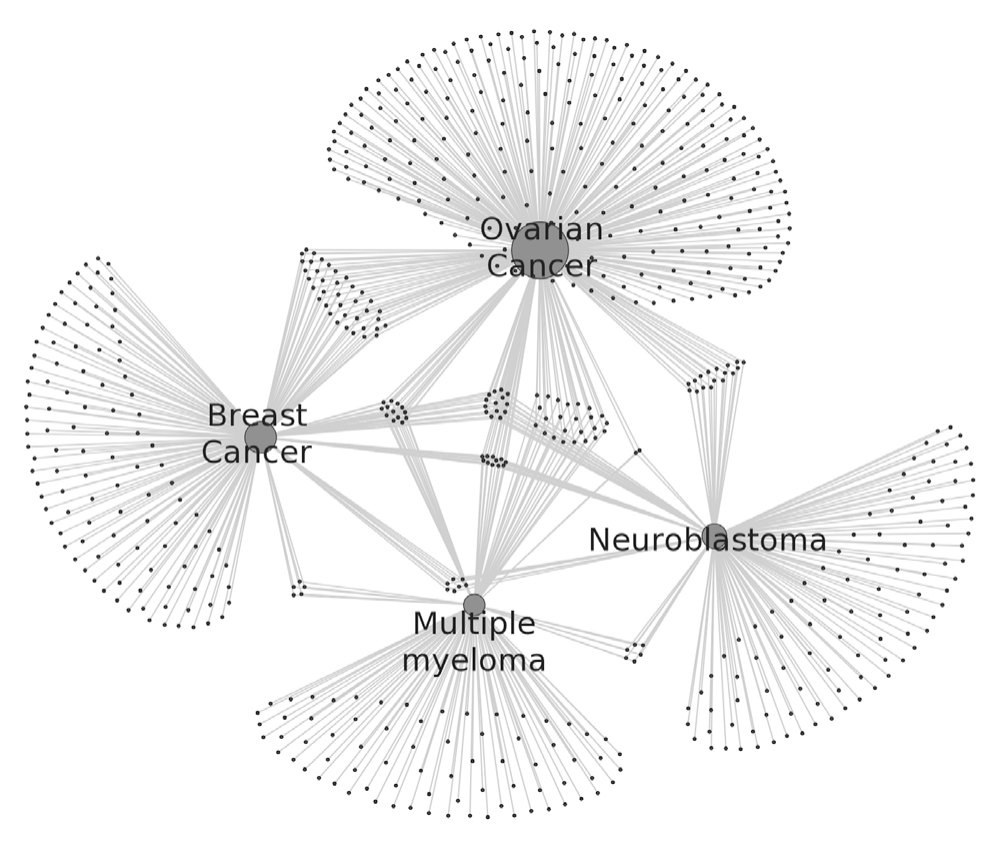
**Genes linked to different types of cancers**. Ovarian, breast cancer, neuroblastoma and multiple myeloma are represented by large grey dots. Genes annotated to each of these diseases are represented by smaller grey dots with 357 genes annotated to ovarian, 199 genes annotated to breast cancer, 156 genes annotated to neuroblastoma, and 135 genes annotated to multiple myeloma. The 11 genes (MMP2, MYC, BCL2, KIT, WT1, CXCL12, CDKN1B, IGF1, CCND1, BIRC5 and SKP2) related to all four diseases are highlighted in the shaded circle at the center.

To illustrate the connected nature of gene-disease mappings, we plot the genes associated with any of the four well-studied cancers (breast, ovarian, neuroblastoma and multiple myeloma) and show the gene-disease relationships from our analysis (Figure 4). Each dark grey dot represents one gene, and 357 genes are annotated to ovarian cancer, 199 genes are annotated to breast cancer, 156 genes are annotated to neuroblastoma, and 135 genes are annotated to multiple myeloma. The diseases are denoted using large light grey dots and the size of the dot is proportional to the number of genes connecting to it. The shaded circle at the center of the figure highlights the 11 genes related to all of four diseases (MYC, BCL2, KIT, WT1, CXCL12, CDKN1B, IGF1, CCND1, BIRC5, SKP2, and MMP2). The functions of these genes include cell cycle regulation, apoptosis, growth factor signaling, and extracellular matrix remodeling. Although these pathways are well known to play a role in cancer initiation or progression, the identification of this specific set of genes may be useful for researcher interested in identifying targets common to these four cancers in particular.

## Discussion

The Disease Ontology consists of a manually inspected subset of UMLS and terms outside the UMLS disease and disorder semantic network including various cancers, congenital abnormalities, deformities and mental disorders that are important to researchers trying to understand the genetic and molecular basis of a particular disease. Therefore, compared to UMLS, the Disease Ontology is much larger in size and more specific to the disease of interest. It therefore offers greater disease coverage with improved accuracy. In addition, its hierarchal structure allows a more specific disease term to be binned to a more general disease term at different levels which is especially useful for Disease Ontology enrichment analysis analogous to gene ontology enrichment analysis in experiments applying high throughput technologies.

Our results indicate that GeneRIFs are an excellent data source for discovering disease-gene relationships. This is primarily due to the large number of GeneRIFs relative to OMIM entries, and the surprisingly high (14.9%) frequency of disease references. The disease coverage of OMIM would be improved if the free text had been mined, but only 235 genes in OMIM have a clinical synopsis section and limiting our analysis to these entries would bias our results. Using the clinical synopsis section in addition to other OMIM free text would increase the number of false positives since OMIM free text frequently includes diseases without a direct relationship to the gene, usually for comparative purposes or in reference to experiments in model organisms.

Errors in our method may arise from a variety of sources including problems with MMTx, many of which have already been elucidated [21]. The problem of having disease terms present in OMIM or GeneRIF, but missing in DO or UMLS was infrequent but did include some cases such Craniofacial-deafness-hand syndrome. A more significant problem contributing to the majority of false positives was the discovery of disease terms in GeneRIF that indicated only a partial, ambiguous or no association to the gene in question. Fortunately, the succinctness of GeneRIF means that this occurs less frequently than in abstracts (data not shown) which may contain diseases not directly related to the gene. We found only one incorrectly assigned GeneRIF in the 1746 GeneRIFs examined, indicating that this is a minor source of error.

One result from our analysis is that OMIM performs poorly relative to GeneRIF with newly discovered mappings. A fairly typical case is the alpha-2-marcroglobuin gene. While OMIM includes mappings for Alzheimer's disease and pulmonary emphysema (missed by GeneRIF), it excludes potential links to benign prostatic hyperplasia, multiple sclerosis and argyrophilic grain disease. This may be a result of OMIM's stronger requirement for evidence, but failure to keep pace with current research may also contribute.

We annotated the human genome with Disease Ontology and reported its performance. Such an annotation will enable many graphical and statistical applications similar to previously what has been done with Gene Ontology annotations. An example of this is presented in Figure 4, where all the genes in the human genome with established links to four cancers led to the identification of eleven genes in common between these four cancers. This analysis facilitates identification of relevant targets or markers for diseases with common etiology or pathology, and has implications for biological plausibility as well as therapeutic potential. Although previous studies have demonstrated the utility of this approach, the improved coverage and accuracy of our analysis provide even greater potential [22]. Our future plans include developing a web-enabled database application of the Disease Ontology for the research community .

## Conclusion

Similar to the GO annotation, we provide a DO annotation of the human genome; each annotation is supported by a peer-reviewed publication as required by GeneRIF. It enables researchers to study gene-disease relationships computationally. The DO annotation of the human genome is available in both tab-delimited format and relational database format , which allows them to be easily adapted for other applications.

## Methods

### MMTx

MMTx is a natural language processing engine that identifies concepts from free text using a lexicon [23]. Briefly, a part-of-speech tagger labels the noun phrases from the lexical elements created after parsing and tokenization (Figure 1A). These noun phrases and variants of these phrases are used to search the UMLS Metathesaurus and outside disease terms to find matching candidates, each of which is given a score; final mappings are generated that best cover the input noun phrase. The Disease Ontology therefore consists of a manually inspected subset of UMLS and terms outside the UMLS disease and disorder semantic network including various cancers, congenital abnormalities, deformities and mental disorders.

Our in-house software parses in the GeneRIF and OMIM data and uses the MMTx API to generate final mappings between genes and diseases. The strict data model of Unified Medical Language System (UMLS) distribution 2005ac was searched against using the default settings of MMTx with an empirically derived score cutoff value of 700. Results were further filtered using the DO version 3.0 (RC9) to eliminate non-disease biological relationships. In addition, a simple heuristic approach was used to eliminate both non-informative mappings to DO (such as "Disease" or "Syndrome") and to eliminate text present in GeneRIF that was frequently mis-mapped by UMLS such as Ca++ ions being mapped to cancer terms. The program calling the MMTx API and generating the gene-disease mapping is written in Java and available upon request.

### GeneRIF and OMIM data

The October 10^th^, 2008 release of both OMIM and GeneRIF were used. For OMIM, only validated data in the formatted "morbidmap" (including disease susceptibilities) file was used. This is because there are currently only 235 records in OMIM which contain a clinical synopsis of the disease, additional disease information is scattered through other sections of the OMIM record making it hard to determine if the disease mentioned is for an animal model or other comparative purposes.

### Scoring and validation

To evaluate our annotation methodology, and to compare our GeneRIF results with the traditional OMIM resource in detail, we utilized a well-characterized fifty-gene collection by Homayouni et al. that they used to evaluate semantic indexing of gene functions [20]. This gene collection includes genes in the reelin signaling pathway of Alzheimer's disease and other genes important in cancer biology and development. We call it Homayouni gene collection from here on. The 5 genes with more than 50 diseases mapped to them (APOE, EGFR, ERBB2, TGFB1 and TP53) were excluded from the test set due to the large number of GeneRIFs requiring manual inspection. This evaluation was done on February 9^th^, 2006.

Assessing the false positive and false negative error rates for this collection was difficult [24], so several domain experts were used for scoring the results with all results reviewed by MID (internal medicine physician) who made the final error determination. To determine gene-disease relationships, a false positive was scored only when the disease was identified incorrectly. No effort was made here to assess the appropriateness of the GeneRIF because of the subjective nature of such a process. However for Table 3 estimates were used for calculating precision and recall rates whereby the overall false positive value was corrected to account for false positives arising when a correctly identified disease did not have a relationship to its associated gene as specified in the GeneRIF.

## Competing interests

The authors declare that they have no competing interests.

## Authors' contributions

JDO, WAK and RLC created the Disease Ontology. The initial annotation of the human genome with DO in 2006 was done by JDO, SML, and WAK; JF updated the annotation and designed the database in 2008. GF and MH conducted the network analysis. MID, LZ, JDO, SML, and WAK evaluated the annotation results using the Homayouni gene collection. JDO drafted the first version of the manuscript and MH worked on the second draft. JDO, SML, WAK and RLC designed the research project. All authors participated in the writing and revision of the paper.
